# Ferredoxin 1b (Fdx1b) Is the Essential Mitochondrial Redox Partner for Cortisol Biosynthesis in Zebrafish

**DOI:** 10.1210/en.2015-1480

**Published:** 2015-12-09

**Authors:** Aliesha Griffin, Silvia Parajes, Meltem Weger, Andreas Zaucker, Angela E. Taylor, Donna M. O'Neil, Ferenc Müller, Nils Krone

**Affiliations:** Centre for Endocrinology, Diabetes, and Metabolism (A.G., S.P., M.W., A.Z., A.E.T., D.M.O., N.K.), School of Clinical and Experimental Medicine (F.M.), College of Medical and Dental Sciences, University of Birmingham, Birmingham B15 2TT, United Kingdom; and Department of Oncology and Metabolism (N.K.), University of Sheffield, Sheffield S10 2TG, United Kingdom

## Abstract

Mitochondrial cytochrome P450 (CYP) enzymes rely on electron transfer from the redox partner ferredoxin 1 (FDX1) for catalytic activity. Key steps in steroidogenesis require mitochondrial CYP enzymes and FDX1. Over 30 ferredoxin mutations have been explored in vitro; however, no spontaneously occurring mutations have been identified in humans leaving the impact of FDX1 on steroidogenesis in the whole organism largely unknown. Zebrafish are an important model to study human steroidogenesis, because they have similar steroid products and endocrine tissues. This study aimed to characterize the influence of ferredoxin on steroidogenic capacity in vivo by using zebrafish. Zebrafish have duplicate ferredoxin paralogs: *fdx1* and *fdx1b*. Although *fdx1* was observed throughout development and in most tissues, *fdx1b* was expressed after development of the zebrafish interrenal gland (counterpart to the mammalian adrenal gland). Additionally, *fdx1b* was restricted to adult steroidogenic tissues, such as the interrenal, gonads, and brain, suggesting that *fdx1b* was interacting with steroidogenic CYP enzymes. By using transcription activator-like effector nucleases, we generated *fdx1b* mutant zebrafish lines. Larvae with genetic disruption of *fdx1b* were morphologically inconspicuous. However, steroid hormone analysis by liquid chromatography tandem mass spectrometry revealed *fdx1b* mutants failed to synthesize glucocorticoids. Additionally, these mutants had an up-regulation of the hypothalamus-pituitary-interrenal axis and showed altered dark-light adaptation, suggesting impaired cortisol signaling. Antisense morpholino knockdown confirmed Fdx1b is required for de novo cortisol biosynthesis. In summary, by using zebrafish, we generated a ferredoxin knockout model system, which demonstrates for the first time the impact of mitochondrial redox regulation on glucocorticoid biosynthesis in vivo.

Steroid hormones are vital to several physiological and developmental processes, including sex development and maintaining homeostasis throughout life. Most enzymatic reactions in the steroidogenic pathway are carried out by cytochrome P450 (CYP) enzymes, which rely on specific redox cofactors to catalyze their oxidative reactions. Although most CYP enzymes are microsomal, CYP type I enzymes are localized in the mitochondria and depend on electron transfer via the ferredoxin-redox system for their hydroxylation activity (1).

Ferredoxins are iron-sulfur (Fe/S) proteins which act as electron donors for a variety of reactions catalyzed by mitochondrial CYP enzymes. The human ferredoxin 1 (FDX1, adrenodoxin, ADX1) is a 14-kDa protein, which is loosely associated with the inner mitochondrial membrane. During electron transfer, the flavoprotein ferredoxin reductase receives electrons from nicotinamide adenine dinucleotide phosphate and in turn reduces FDX1. FDX1 transfers these electrons to the respective CYP enzymes allowing them to perform their catalytic functions (1). Humans have 7 CYP type I enzymes which are involved in metabolic processes, including the biosynthesis of steroid hormones (2) and bile acid (CYP27A1), and vitamin D metabolism (CYP24A1 and CYP27B1) (3). In steroidogenic tissues, FDX1 transfers electrons to the P450 side-chain cleavage enzyme (CYP11A1), which facilitates the conversion of cholesterol into pregnenolone as the first and rate-limiting step of steroid hormone biosynthesis (4). CYP11A1 catalyzes 3 sequential monooxygenase reactions; the 2 hydroxylation reactions of cholesterol generate 22R-hydroxycholesterol and 20α,22R-dihydroxycholesterol and the final cleavage of the bond between carbons 20 and 22 to generate pregnenolone. For each catalytic step CYP11A1 requires 2 electrons from FDX1, making FDX1 an important component in the regulation of steroid hormone biosynthesis. In addition, the mitochondrial CYP enzymes 11β-hydroxylase (CYP11B1) and aldosterone synthase (CYP11B2) also require ferredoxin reductase/FDX1 electron transfer to catalyze the final reactions for glucocorticoid and mineralocorticoid biosynthesis, respectively (5). As these reactions are essential for glucocorticoid and mineralocorticoid production it is imperative that the activity of these enzymes is tightly regulated to maintain normal physiology. In vitro studies suggest that modifying FDX1 concentrations or FDX1 mutants harboring different redox potentials can influence CYP catalytic activity (6, 7). However, the influence of ferredoxin electron transfer on normal in vivo steroid hormone biosynthesis remains unknown as there are currently no *Fdx1* knockout animal models or human mutations identified.

Zebrafish (*Danio rerio*) are a popular vertebrate model to elucidate gene function in development and disease. Despite the evolutionary distances between mammals and fish, the molecular pathways controlling glucocorticoid biosynthesis, the developmental programming, and physiological actions of glucocorticoid signaling remain highly conserved (8, 9). Similar to humans, cortisol is the main glucocorticoid in zebrafish. It is produced in the interrenal gland, which is the equivalent of the mammalian adrenal gland (9, 10). The interrenal gland is located within the anterior part of the kidney head and produces cortisol under the regulation of the hypothalamus and pituitary, forming the hypothalamus-pituitary-interrenal (HPI) axis (11–13). Mitochondrial electron transfer cofactors involved in steroidogenesis, such as FDX1, are also well conserved in zebrafish. Zebrafish have 2 *FDX1* paralogs termed *fdx1* and *fdx1b*. To date, the characterization of these paralogs and their relevance to cortisol biosynthesis has not been reported. Therefore, the aim of this current study was to define the role of the zebrafish *fdx1* paralogs in glucocorticoid biosynthesis and to establish an in vivo model to explore the role of ferredoxin in regulating steroidogenic capacity. Here, we describe that Fdx1b is the essential mitochondrial redox partner required for glucocorticoid biosynthesis and it is essential for de novo steroidogenesis in zebrafish.

## Materials and Methods

### Alignment and phylogenetic analyses of zebrafish Fdx isozymes

Zebrafish Fdx1 and Fdx1b protein sequences were compared with vertebrate Fdx1 protein sequences publicly available at the Ensembl Genome Browser (Ensembl protein ID), including human (*Homo sapiens*; ENSP00000260270), gorilla (*Gorilla gorilla gorilla*; ENSGGOP00000023526), chimpanzee (*Pan troglodytes*; ENSPTRP00000007328), bovine (*Bos taurus*; ENSBTAP00000015660), rabbit (*Oryctolagus cuniculus*; ENSOCUP00000015307), mouse (*Mus musculus*; ENSMUSP00000034552), rat (*Rattus norvegicus*; ENSRNOP00000016263), medaka (*Oryzias latipes*; ENSORLP00000005248), tetraodon (*Tetraodon nigroviridis*; ENSTNIP00000013263), and stickleback (*Gasterosteus aculeatus*; ENSGACP00000018745). Alignment and homology analysis were performed using ClustalW2 software under default parameters (http://www.ebi.ac.uk/Tools/msa/clustalw2/). Phylogenetic analysis of protein sequences were performed with the PhyML software under the SH-like likelihood-ratio test parameters (http://www.phylogeny.fr/) (14). A scaled phylogenetic tree was drawn with TreeDyn software.

### RT-PCR expression analysis of *fdx1* and *fdx1b*

Pools of 10 zebrafish embryos or larvae were collected at 1-cell, 4-cell, 64-cell, 1000-cell, sphere, 50% epiboly, 70% epiboly, 10, 22–24, 48, 52, 72, 96, and 120 hours postfertilization (hpf) in triplicate for expression analysis. Zebrafish gonads, brain, kidney head, posterior kidney, heart, intestine, and liver were dissected from 3 female and 3 male adult zebrafish euthanized in compliance with the Home Office Animal Licensing Act 1986.

Total mRNA was extracted from whole embryos or larvae and adult tissues using TriReagent (Sigma-Aldrich Ltd) according to the manufacturer's protocol. After extraction, 1 μg of mRNA was incubated with 1-U DNaseI amplification grade (Life Technologies) at 37°C for 1 hour, followed by DNaseI inactivation with 2mM EDTA for 15 minutes at 65°C. Purified mRNA was retrotranscribed to cDNA using 1-μL SuperScriptIII and 2.5μM oligo(dT)_20_ primers (Sigma-Aldrich) following the manufacturer's protocol.

The temporal expression of *fdx1* and *fdx1b* was characterized in triplicate by RT-PCR. A 694-bp fragment of *fdx1* and a 159-bp fragment of *fdx1b* were amplified using MegaMix-Blue reaction mix (Microzone Ltd) containing 200nM primers and 25 ng of cDNA under the next conditions: initial denaturation at 95°C for 5 minutes, followed by 40 cycles at 95°C for 30 seconds, 58°C for 40 seconds, and 72°C for 60 seconds and a final incubation at 72°C for 7 minutes. As a control for cDNA quality, a 102-bp fragment of the β-actin gene (*actb1*; ENSDARG00000037746) was also amplified. Oligonucleotide sequences are listed in Supplemental Table 1.

### Whole-mount in situ hybridization (WISH)

Spatial expression in larvae was determined by WISH. Standard WISH were carried out as previously described (15) at 120 hpf using digoxigenin-labeled RNA probes. Riboprobe-generating sequences were amplified from cDNA using primers specific for *fdx1* (forward, 5′-TGCGTGTGTTTTAAGAGCGT-3′ and reverse, 5′-ACCAGATGAGTGTTGCAGAA-3′) and *fdx1b* (forward, 5′-ACAGGAACGTTTTATGCCCG-3 and reverse, 5′-TCACCTGACAACCCAATCGA-3′).

### Genotyping *fdx1b* mutants by high-resolution melting-curve (HRM) analysis

HRM analysis was performed using 7900HT Fast Real-Time PCR system (Applied Biosystems) using 384-well block module. Ten-microliter reactions were performed in duplicate containing 1× SYBRGreen Master Mix (Applied Biosystems), 150nM primers (forward, 5′-CTATATTAGGAGCATGCGAGG and reverse, 5′-CATGTCAATCTCTTCATCCACC) and 3 μL of gDNA. An initial holding stage at 95°C for 10 minutes was followed by 40 cycles of denaturing at 95°C for 15 seconds and annealing and extending at 60°C for 1 minute. The dissociation phase consisted of denaturing at 95°C for 10 seconds, annealing and extension for 1 minute at 60°C, high-resolution melting at 95°C for 15 seconds with a ramp rate of 1% and an annealing step of 60°C for 15 seconds. Amplification was confirmed by analysis of cycle threshold (C_t_) values. To detect indels, changes in the dissociation curve were compared with wild-type controls and confirmed by sequencing.

### Visual background adaptation (VBA) assay

To identify larvae defective in VBA behavior, clutches were subjected to the VBA assay at 96 or 120 hpf. Briefly, larvae were maintained in a dark environment for at least 45 minutes, followed by exposure under fluorescent illumination on a white background for 20 minutes.

### Zebrafish *fdx1* and *fdx1b* knockdown studies

Antisense morpholinos (GeneTools LLC) were used for transient gene knockdown. Translation blocking and splice disrupting morpholinos were designed to specifically target *fdx1* (*fdx1*-ATG^MO^ and *fdx1*-Spl^MO^) or *fdx1b* (*fdx1b*-ATG^MO^ and *fdx1b*-Spl^MO^). Five-base pair mismatch for the ATG morpholinos were used as controls (*fdx1*-Ctl^MO^ and *fdx1b*-Ctl^MO^). Sequences of the morpholinos are listed in Supplemental Table 2. Morpholinos were diluted to working concentration 1.5–4.5 ng/nL in sterile water containing 0.05% phenol red and 5% rhodamine B (Sigma-Aldrich). Two nanoliters of working solution was microinjected at the 1-cell stage. Microinjection of control morpholinos at the same concentration did not show off-target effects. Rhodamine B was used to screen for injected embryos using a 554 nm laser on a fluorescence microscope (SMZ1500-P-FLA2; Nikon UK Limited). Abnormal splicing of the *fdx1b* gene in *fdx1b*-Spl morphants was tested by RT-PCR using *fdx1b* primers listed in Supplemental Table 1. To generate *fdx1* and *fdx1b* mRNA, coding sequences were cloned into pCS2+ expression vector. Fdx1 was PCR amplified containing 5-bp synonymous mutations within the morpholino binding site and included a 5-prime T7 polymerase recognition sequence and a 3-prime polyadenylation signal. Capped mRNA was synthesized in vitro direct from the purified PCR product using T7 mMessage mMachine kit (Life Technologies); 50-pg/nL mRNA was added to the morpholino injection mix. Embryos injected with *fdx1*-ATG^MO^, *fdx1*-Spl^MO^, or *fdx1*-Ctl^MO^ were classified based on their progression of epiboly at 9 hpf. Class I represents a normal epiboly, and class II and class III represent increase severity of delayed movement.

### Steroid measurements in zebrafish Fdx1b-deficient embryos

A total of 300 morpholino-injected zebrafish were collected at 10, 22–24, 72, 96, and 120 hpf. Alternatively, 200 *fdx1b* mutants or wild-type siblings were collected at 120 hpf. Dechorionated embryos and larvae were washed twice in 1× Dulbecco's modified PBS (Sigma-Aldrich) and kept in 1-mL 1× Dulbecco's modified PBS at −20°C until required. Steroid hormones were extracted as previously described (16) and detailed in Supplemental Methods. Steroid measurements are expressed in picograms per larva ± SD and plotted using GraphPad Prism v.5.0 software (GraphPad Software, Inc).

### Treatments of zebrafish larvae

For steroid hormone treatment, ten larvae were incubated overnight in embryo (E3) media containing either 100μM dexamethasone or 0.1% ethanol. The next day, larvae were assessed for VBA ability. For osmotic stress induction, larvae were subjected to 250mM NaCl for 20 minutes using a standard protocol (17). Subsequently, larvae were washed and incubated in 1× E3 media for 4 hours before sampling. For treatment of morphants, *fdx1b*-ATG^MO^-injected embryos were supplemented with 50nM pregnenolone or 0.1% ethanol from 10 hpf. Treatments were changed daily, and at 72 hpf, embryos were collected for steroid extraction.

### Quantitative real-time mRNA analysis

The expression of proopiomelanocortin (*pomca*) and the housekeeping gene glyceraldehyde 3-phosphate dehydrogenase (*gapdh*) was determined using an ABI 7500 sequence detection system (Applied Biosystems). Reactions were performed in 20-μL volumes on 96-well plates using TaqMan Universal PCR Master Mix and expression-specific TaqMan Gene Expression Assays (Life Technologies). Analysis of phosphoenolpyruvate carboxykinase 1 (*pck1*) and FK506-binding protein 5 (*fkbp5*) (18) as well as *fdx1* and *fdx1b* were performed using SYBRGreen Master Mix (Applied Biosystems), with 150nM primer and 2 μL of cDNA. Oligonucleotide sequences are listed in Supplemental Table 1. Experiments were analyzed from 3 independent experiments. Data were expressed as C_t_ values and used to determine ΔC_t_ values.

### Statistical analysis

For the analysis of independent-samples *t* tests were used as appropriate for comparison between 2 groups. One-way ANOVA after either Dunnett's multiple comparison test for analysis against a control sample or Holm-Sidak pairwise multiple comparisons between means. Differences considered statistically significant are indicated with asterisks (*, *P* < .05; **, *P* < .01; ***, *P* < .001).

## Results

### Zebrafish Fdx1 and Fdx1b share homology with other vertebrate Ferredoxins

**Figure 1.**
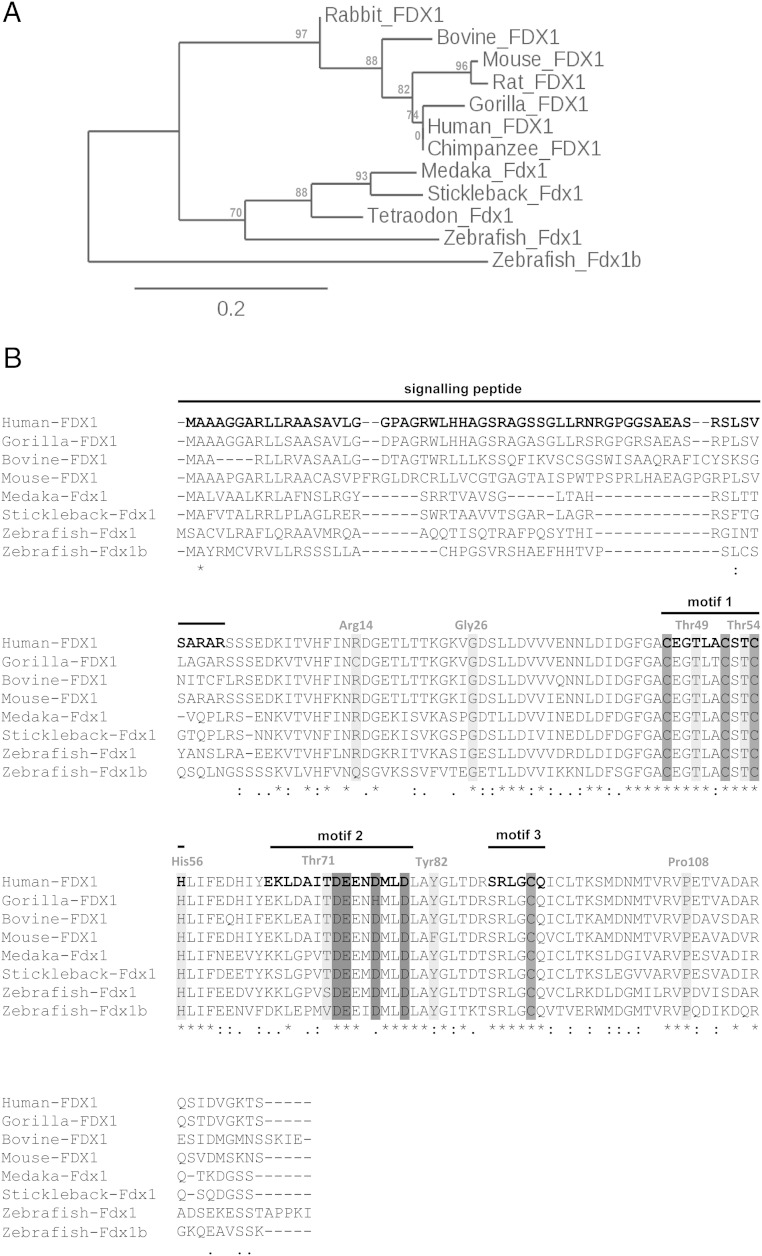
Evolutionary conservation of vertebrate ferredoxins. A, Maximum likelihood phylogenetic analysis of vertebrate Fdx1 protein sequences were conducted using the PhyML software under the SH-like likelihood-ratio test parameters. Scaled phylogenetic tree was drawn with TreeDyn software. Branch support values are represented in % and shown in red. B, The preprotein sequence of human, gorilla, bovine, mouse, medaka, stickleback, and zebrafish ferredoxin enzymes were aligned using ClustalW under default parameters. The 3 motifs common to all hydroxylase ferredoxins are shown. Motif 1 is a loop that contains 3 of the cysteine residues involved in Fe/S cluster binding (gray). Motif 2 contains a group of negatively charged amino acid residues, which are important for binding to CYP and ferredoxin reductase (dark gray). Motif 3 contains the conserved sequence around the fourth cysteine residue required for cluster binding. The signaling peptide targets the ferredoxin to the mitochondria and is cleaved to form the mature protein. Other known functionally important amino acid residues required for protein stabilization and redox potential are shown in gray. Amino acid residues are numbered according to the mature human FDX1 sequence.

To determine the evolutionary conservation of the 2 zebrafish ferredoxin paralogs *fdx1* and *fdx1b*, protein sequence analysis was performed in silico. Initially, the *fdx1* and *fdx1b* coding sequences were amplified and sequenced from cDNA extracted from ovary of an adult female and nucleotide sequences were used to infer protein sequences. Zebrafish Fdx1 and Fdx1b are 175- and 168-amino acid residues in length and share 43% amino acid sequence identity with Fdx1 being most similar to other teleost species (Figure 1A). Importantly, high conservation in functional motifs was observed in both paralogs. Motif 1 and motif 3 collectively contain the 4 cysteine residues required for Fe/S binding (19) and are present in both zebrafish paralogs. Also, the negatively charged motif 2 containing amino acid residues required for the interaction with redox partners is conserved. Other functionally important amino acid residues are also present and noted in Figure 1B.

### Expression of *fdx1* and *fdx1b* genes

**Figure 2.**
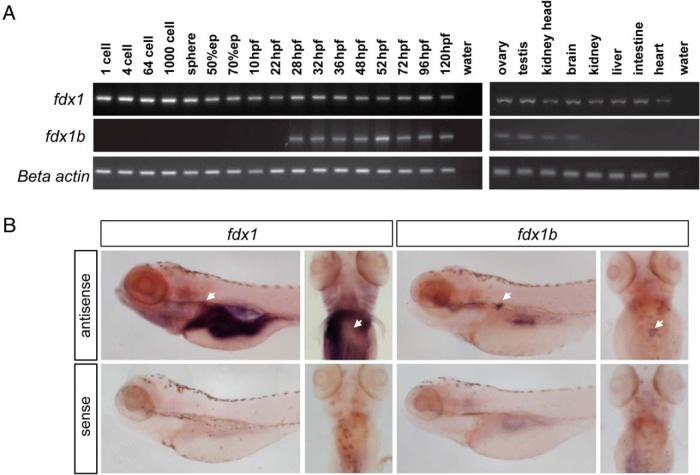
Temporal and spatial expression of zebrafish *fdx1* paralog genes. A, Expression of *fdx1* and *fdx1b* was characterized in zebrafish during development and in adult tissues. Representative agarose gel images are shown from triplicate experiments from pools of 10 zebrafish embryos or larvae at the specified developmental stages or from adult tissues. β-Actin was used as a control for integrity of template cDNA. B, Detection of *fdx1* and *fdx1b* mRNA at 120 hpf by WISH. Lateral and dorsal views are shown. Negative control sense probes did not show staining. Arrow indicates location of the interrenal gland.

The expression of *fdx1* and *fdx1b* genes was investigated by RT-PCR during early zebrafish development and in adult fish (Figure 2A). *fdx1* was expressed as a maternal transcript (present from 1-cell stage) and was maintained by the zygote throughout development. Expression of *fdx1b* started during late segmentation period (28 hpf) and was maintained to 120 hpf. In adults, *fdx1* expression was detected in all tissues tested. Zebrafish *fdx1b* expression was identified in tissues with established steroidogenic activity, including the kidney head (containing the interrenal gland), brain, ovary, and testis. No sexual dimorphism in the expression pattern of either gene was observed. Spatial expression by WISH at 120 hpf, showed *fdx1b* has high expression within the interrenal cells of the kidney head, whereas *fdx1* shows wider expression pattern with high staining in the liver, intestine, and gut (Figure 2B).

### Gene targeting of *fdx1b* in zebrafish by transcription activator-like effector nucleases (TALENs)

**Figure 3.**
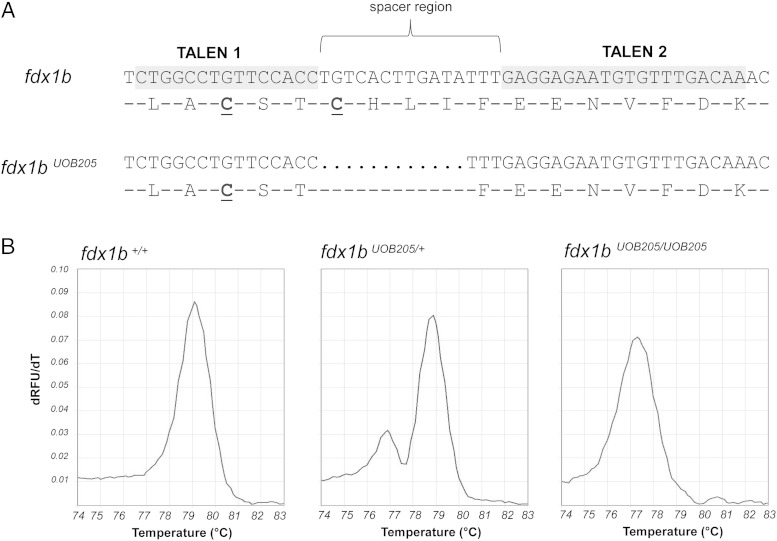
Genetic disruption of the *fdx1b* locus in zebrafish by TALENs. A, Exon 4 of *fdx1b* was targeted for genetic disruption by TALENs. TALEN1 and TALEN2 recognized 15 and 20 nucleotides, respectively (gray). Each TALEN was separated by a 15-nucleotide spacer region. A line harboring a 12-bp deletion was established (*fdx1b^UOB205^*) (c.295_306del; p.Cys99_Ile102del). This mutation resulted in a 4-amino acid deletion, which includes a cysteine essential for Fe/S cluster binding (underlined). B, Genotyping of individual embryos was performed by HRM analysis. PCR amplification of wild-type *fdx1b* (*fdx1b*^+/+^) gives a product with a melting peak at 79°C, whereas *fdx1b^UOB205^* allele gives a product with a melting peak at 77°C. Representative melting peaks are shown for *fdx1b*^+/+^, *fdx1b* heterozygous (*fdx1b*^*UOB205*/+^), and embryos homozygous for the mutation (*fdx1b^UOB205/UOB205^*).

Given the restricted expression of *fdx1b* to steroidogenic tissues, we further investigated its role by disrupting the *fdx1b* gene using TALENs. TALENs allow for targeted gene disruption and permit the generation of heritable mutations for loss-of-function studies. *fdx1b* TALEN binding sites were chosen within exon 4, which encodes the functionally conserved motif 1. Specifically, the left TALEN targeted 15 nucleotides of *fdx1b* and the right TALEN targeted 20 nucleotides. Each was separated by 15-nucleotide spacer sequence (Figure 3A). Genomic disruption in injected embryos was confirmed by HRM analysis. The germ line transmission rate of indels was 27% (4 founders out of 20 adults screened). Sequencing identified 4 different heritable alleles within the targeted spacer region (Supplemental Figure 1). Subsequently, we established a mutant allele (allele *fdx1b^UOB205^*) with a 12-bp deletion leading to a 4-amino acid in-frame deletion (c.295_306del; p.Cys99_Ile102del). This mutation removes a conserved cysteine residue required for stabilizing the Fe/S cluster (Figure 3A). HRM analysis was used to confirm the identification of wild-type, heterozygous, and *fdx1b^UOB205^* homozygous mutant larvae (Figure 3B). A second line harboring a 7-bp indel generated at the same locus (allele *fdx1b^UOB203^*) caused a frame-shift that is predicted to remove motifs 2 and 3 (c.294_305delinsTTGTC; p.Ile102fs*34) (Supplemental Figure 2A). Homozygous mutants were obtained from heterozygous in-crosses and maintained to 5 days postfertilization and no deviation from Mendelian ratios was observed (ie, the ratio among wild-type to heterozygous to homozygous mutants was 1:2:1).

### Glucocorticoid deficiency in *fdx1b* mutant zebrafish

**Figure 4.**
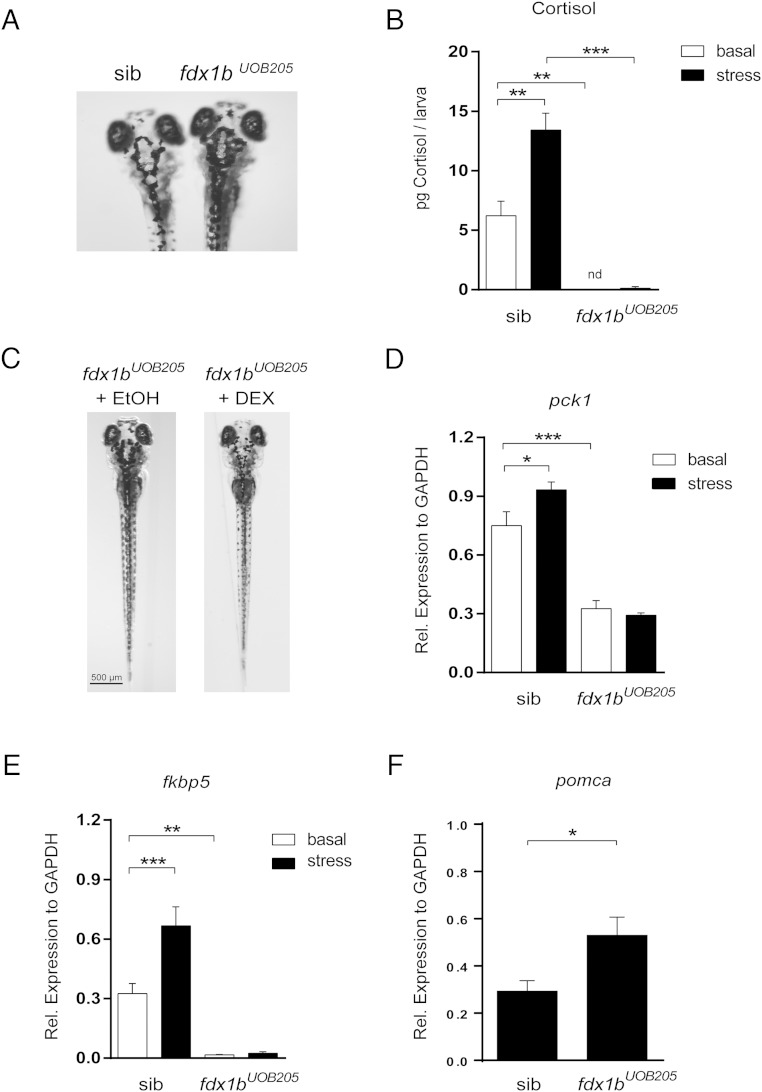
Zebrafish *fdx1b^UOB205^* homozygous mutants are glucocorticoid deficient. A, *fdx1b^UOB205^* homozygous mutants have impaired visual-mediated adaption. When exposed to a lighter environment, *fdx1b^UOB205^* homozygous mutants are darker in appearance when compared with control siblings, which had a least 1 wild-type allele (*fdx1b*^+/+^ or *fdx1b*^*UOB205*/+^). B, Cortisol concentrations were determined from *fdx1b^UOB205^* homozygous mutant larvae (*fdx1b^UOB205^*) and sibling controls (sib) under basal and stressed conditions. Cortisol was not detected (nd) from *fdx1b^UOB205^* homozygous mutants under basal conditions. Concentrations were determined from 3 independent replicates and are expressed as picograms per larva (mean ± SD). C, Treatment of *fdx1b^UOB205^* homozygous mutants with the glucocorticoid agonist dexamethasone (DEX) restored the light adaptation phenotype in contrast to ethanol-treated controls (EtOH). mRNA expression of glucocorticoid response genes (D) *pck1* and (E) *fkbp5* in *fdx1b^UOB205^* homozygous mutants and control siblings under basal and stressed conditions. Graphs represent mean relative expression ± SEM (normalized to *gapdh*). F, Quantitative real-time expression of *pomca* in *fdx1b^UOB205/UOB205^* mutants relative to control siblings. Statistical analysis was performed using one-way ANOVA; *, *P* < .05; **, *P* < .01; ***, *P* < .001.

To determine the requirement of Fdx1b for steroid hormone production, we characterized homozygous *fdx1b* mutants during the first 5 days of zebrafish development. *fdx1b^UOB205^* homozygous mutants were morphologically similar to control siblings during this time. Because background adaptation in zebrafish has been associated with glucocorticoid signaling (20), we subjected larvae to VBA assessment. Larvae from 2 different clutches from an *fdx1b^UOB205^* heterozygous in-cross were subjected to VBA assessment at 120 hpf and sorted into dark pigmentation (22.8%; n = 33) and light pigmentation (77.2%; n = 112) (Figure 4A). Genotyping of 20 fish with impaired VBA revealed larvae were homozygous for the *fdx1b^UOB205^* allele. Larvae capable of light adaptation were either *fdx1b* wild-type (*fdx1b*^+/+^) or *fdx1b* heterozygous (*fdx1b*^*UOB205*/+^), confirming *fdx1b* homozygous mutants can reliably be identified from their siblings by VBA assessment.

At 120 hpf, larvae from a heterozygous in-cross were genotyped by VBA and collected for steroid hormone measurements. Cortisol concentrations were undetectable in *fdx1b^UOB205^* homozygous mutant larvae under normal conditions. In contrast, light adapted control siblings had 6.2 pg of cortisol per larva. Under osmotic stress, cortisol was increased to 13.4 pg per larva for *fdx1b* sibling controls. However, under the same conditions *fdx1b^UOB205^* homozygous mutants had 0.3 pg per larva (Figure 4B). The impairment of cortisol biosynthesis at 120 hpf was confirmed in *fdx1b^UOB203^* homozygous mutants representing a second mutant line (Supplemental Figure 2B). To confirm that the VBA phenotype in *fdx1b* homozygous mutant larvae was being mediated by defective cortisol production, 15 larvae with compromised VBA were identified at 96 hpf and treated overnight with dexamethasone or vehicle control. VBA reassessment at 120 hpf revealed that dexamethasone treatment was able to restore light adaptation in all larvae (Figure 4C). Genotyping of dexamethasone and control treated zebrafish confirmed larvae were *fdx1b^UOB205^* homozygous mutants.

To further assess how the loss of *fdx1b* affects glucocorticoid signaling and action, quantitative RT-PCR was used. Expression of the glucocorticoid responsive genes *pck1* and *fkbp5* was decreased in *fdx1b^UOB205^* homozygous mutants compared with sibling controls under normal conditions. Expression levels were unchanged in *fdx1b^UOB205^* homozygous mutants when larvae were subjected to osmotic stress, suggesting impaired glucocorticoid stress response. In comparison, increased expression of both genes was observed in *fdx1b* sibling controls when subjected to osmotic stress. Increased expression of pituitary derived *pomca* was also observed in *fdx1b^UOB205^* homozygous mutants compared with *fdx1b* control siblings, indicating activation of the HPI axis due to deficient glucocorticoid synthesis (Figure 4, D–F). No change in expression levels of either ferredoxin gene was observed between *fdx1b^UOB205^* homozygous mutants and sibling controls under basal conditions. This suggests no compensatory increase of *fdx1* expression in *fdx1b* mutants (Supplemental Figure 3).

### Disrupted steroid biosynthesis in *fdx1b* mutant zebrafish

**Figure 5.**
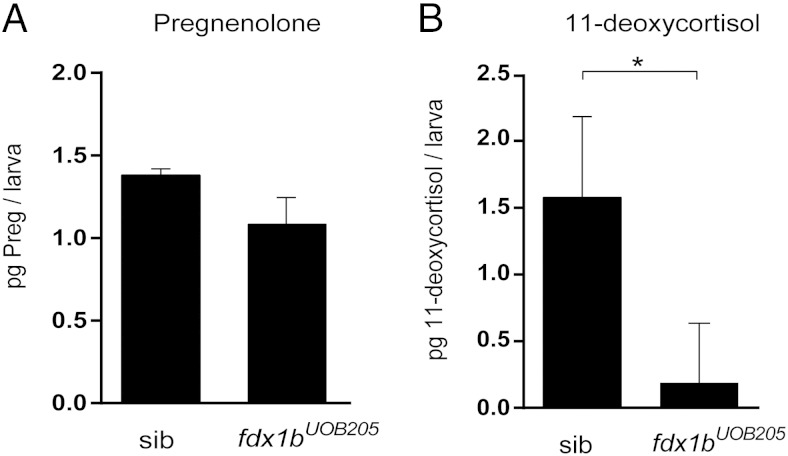
Pregnenolone and 11-deoxycortisol measurements in zebrafish *fdx1b^UOB205^* homozygous mutants. A, Pregnenolone concentrations were measured in *fdx1b^UOB205^* homozygous mutants (*fdx1b^UOB205^*) and sibling controls (sib) under normal conditions at 120 hpf. B, 11-deoxycortisol was measured after stress induction in mutants and controls. Steroid profiles were determined from 3 independent replicates and represented as mean picograms per larva ± SD. Statistical significance was determined by Student's *t* test; *, *P* < .05.

To assess the importance of Fdx1b on the steroid biosynthesis pathway, we measured pregnenolone and 11-deoxycortisol by liquid chromatography tandem mass spectrometry in *fdx1b* homozygous mutants. Under normal conditions the *fdx1b^UOB205^* homozygous mutants were detected having 1.1 pg of pregnenolone per larva compared with *fdx1b* sibling controls which had 1.4 pg pregnenolone per larva (Figure 5A). Comparable pregnenolone concentrations were observed in a second line, *fdx1b^UOB203^* homozygous mutants, which had 1.1 pg of pregnenolone per larva compared with 1.3 pg per *fdx1b* control sibling (Supplemental Figure 2C). The glucocorticoid intermediate 11-deoxycortisol was also analyzed in stressed *fdx1b^UOB205^* homozygous mutants. At 120 hpf, *fdx1b* sibling controls had 1.6 pg per larva which was significantly decreased in *fdx1b^UOB205^* homozygous mutants with 0.2 pg of 11-deoxycortisol per larva (Figure 5B). This suggests upstream impairment of de novo 11-deoxycortisol production in *fdx1b* mutants.

### The impact of Fdx1b of steroidogenesis during early zebrafish development

**Figure 6.**
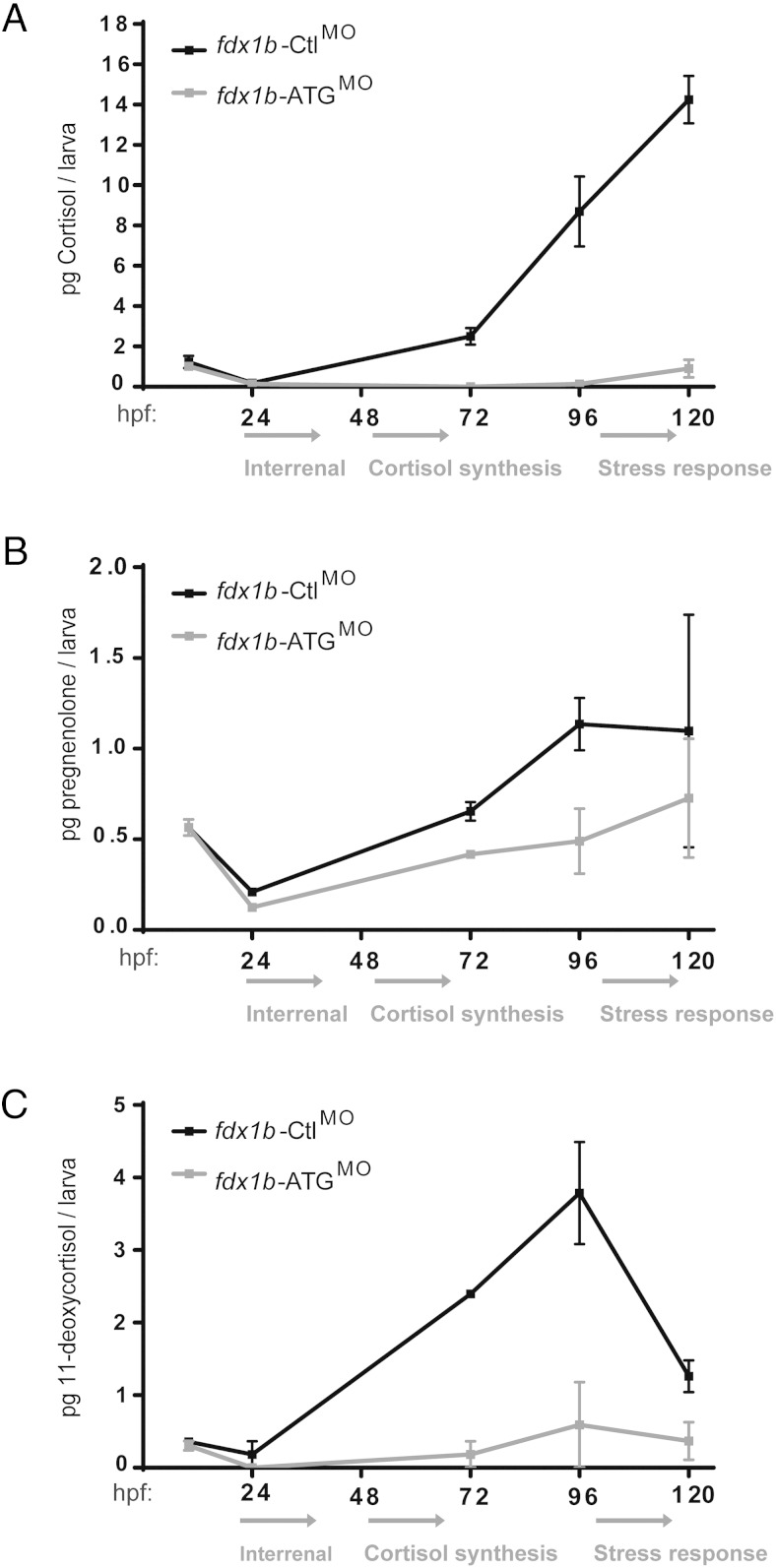
Fdx1b deficiency impairs de novo steroidogenesis during zebrafish development. Cortisol (A), pregnenolone (B), and 11-deoxycortisol (C) concentrations were measured during development from pools of 300 embryos injected with either *fdx1b*-ATG^MO^ (gray) or *fdx1b*-Ctl^MO^ (black). Picograms of steroid per embryo or larva were calculated from concentrations measured from whole zebrafish extracts. Experiments were performed in duplicate. Mean ± SD was plotted against developmental stages.

To identify the in vivo role of *fdx1b* during early zebrafish development and confirm the *fdx1b* mutant phenotype, cortisol concentrations were measured in *fdx1b-*ATG^MO^ and *fdx1b-*Ctl^MO^-injected embryos across 5 developmental stages. At 10 and 24 hpf, there was no difference in cortisol concentrations between *fdx1b*-deficient and control embryos. At 72 hpf, cortisol was not detected in *fdx1b-*ATG morphants, whereas control larvae had 2.5 pg at this time point. At 96 and 120 hpf, *fdx1b* morphants had 0.1 and 0.9 pg of cortisol per larva, respectively, compared with 8.7 and 15.3 pg per larva in the control group (Figure 6A). A second splice morpholino (*fdx1b*-Spl^MO^) was used to confirm gene disruption by RT-PCR. Embryos injected with the *fdx1b*-Spl^MO^ had cortisol concentrations of 0.4 pg per embryo at 72 hpf (Supplemental Figure 4, A–C).

In addition to cortisol, pregnenolone and 11-deoxycortisol concentrations were also measured in morphants to determine the requirement of Fdx1b for steroid hormone biosynthesis during development. No difference was observed between *fdx1b*-ATG morphants and controls in either metabolite at 10 or 24 hpf. At 72 hpf, *fdx1b*-ATG morphants had pregnenolone concentrations of 0.4 pg per larva compared with 0.7 pg per larva in controls. These morphants maintained lower pregnenolone concentrations at 96 and at 120 hpf were detected having 0.7 pg in contrast to the control-injected embryos with 1.1 pg (Figure 6B). At 72 and 96 hpf *fdx1b*-ATG morphants had 0.2 and 0.6 pg of 11-deoxycortisol per larva, respectively. In comparison control morphants had significantly higher 11-deoxycortisol concentrations of 2.4 and 3.8 pg per larva for the same time points. At 120 hpf, 11-deoxycortisol concentrations decreased in control-injected larvae to 1.3 pg per larva, whereas *fdx1b*-ATG morphants showed 0.4 pg per larva (Figure 6C).

**Figure 7.**
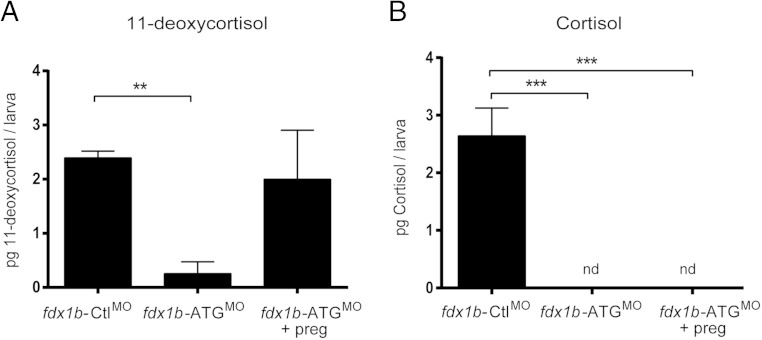
Pregnenolone supplementation rescues 11-deoxycortisol but not cortisol production in *fdx1b*-deficient zebrafish at 72 hpf. *fdx1b*-ATG morphants were incubated in E3 medium containing 50nM pregnenolone from 10 hpf. 11-deoxycortisol (A) and cortisol (B) concentrations were measured in 72-hpf zebrafish larvae from 3 independent experiments. Concentrations expressed as picograms per larva were calculated from concentrations measured from whole zebrafish extracts. Mean ± SD is plotted against the treatment and compared using one-way ANOVA; ***, *P* < .001. nd, not detected.

To further elucidate the role of Fdx1b in zebrafish steroidogenesis, we supplemented zebrafish *fdx1b*-ATG^MO^ with pregnenolone and measured 11-deoxycortisol and cortisol at 72 hpf. Pregnenolone restored 11-deoxycortisol concentrations to 2.0 pg per larva, which was comparable with control-injected larvae. Cortisol remained undetectable in *fdx1b* morphants supplemented with pregnenolone, suggesting severe impairment of the final 11β-hydroxylation step of glucocorticoid biosynthesis (Figure 7).

### *fdx1* knockdown leads to a severe impairment of early zebrafish development

**Figure 8.**
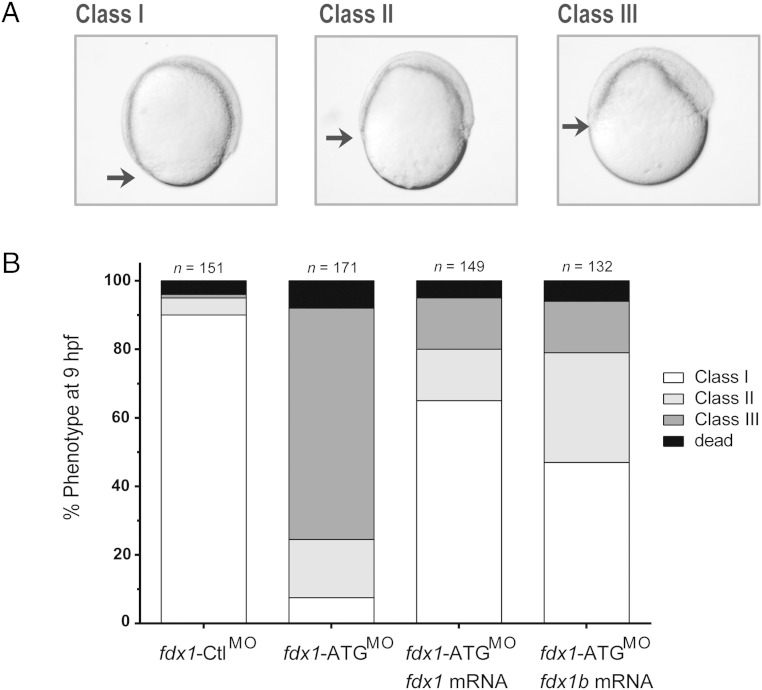
Morphological phenotype of embryos injected with *fdx1* morpholino at 9 hpf. A, Embryos injected with *fdx1*-ATG^MO^, *fdx1*-Spl^MO^, or *fdx1*-Ctl^MO^ were classified based on their progression of epiboly at 9 hpf. Class I represents a normal epiboly, and class II and class III represent delayed movement as indicated by the black arrow. B, Injection of 9 ng of *fdx1*-Ctl^MO^ showed 95% of the normal class I phenotype compared with *fdx1*-ATG^MO^-injected embryos, which had 68.9% class II and 18% class III. Coinjection of either *fdx1* or *fdx1b* mRNA with *fdx1*-ATG^MO^ partially restored embryos to a class I phenotype.

To determine the impact of Fdx1 loss of function on steroidogenesis during zebrafish development transient knockdown, studies were performed using morpholinos. Microinjection of the *fdx1*-ATG^MO^ resulted in a phenotype during the gastrula phase. By 9 hpf, 69% of *fdx1* morphants showed a class II phenotype consisting of a delay in epiboly (the movement of cells over the yolk during gastrulation) and a thickening of the dorsal blastoderm. Additionally, 18% of *fdx1* morphants showed a class III phenotype, where a severe delay in epiboly was observed, together with a grainy appearance of the cells of the blastoderm. In contrast, 97% of control-injected embryos showed a typical class I phenotype with normal developmental progression through epiboly (Figure 8). Severity of the phenotype increased with increasing concentrations of morpholino (Supplemental Figure 5) and microinjection of *fdx1-*Spl^MO^ partially reproduced the phenotype with 40% of embryos showing wither class II, class III, or failing to thrive by 9 hpf (data not shown). Coinjection with full-length *fdx1* mRNA or *fdx1b* mRNA with the *fdx1*-ATG^MO^ was able to restore 65% and 47% of embryos, respectively, to a normal class I developmental phenotype (Figure 8). Because the knockdown of *fdx1* leads to an early severe gastrulation phenotype, further studies on the effects on interrenal steroidogenesis were not possible.

## Discussion

### Evolutional conservation of ferredoxin electron transport system

The evolutionary conservation of ferredoxins is well established (3). We observed both Fdx1 and Fdx1b show conservation of important amino acids, including the histidine 56 and proline 108, which are present in all vertebrate ferredoxins (6), and threonine residues at positions 49 (21) and 54 (22), which are both involved in modulating the redox potential. Despite having evolutionary well-conserved functional domains, the zebrafish ferredoxin preproteins share 43% amino acid sequence identity, with differences which may provide insights into the structural and functional relationships of ferredoxins.

Posttranslational in vitro modifications of ferredoxins can alter activities of the interacting CYP enzymes. In vitro, phosphorylation of bovine Fdx1 at position threonine 71 increases CYP11A1 enzymatic activity, but not CYP11B1 activity (7), suggesting an important regulatory mechanism impacting on mitochondrial CYP enzyme function. Interestingly, zebrafish Fdx1 has a serine at this position, which is capable of being phosphorylated, whereas Fdx1b has a valine residue, which is not amenable to phosphorylation. However, the mechanistic importance of posttranscriptional modification, including phosphorylation of redox partners during in vivo steroidogenesis, has not yet been elucidated. The biochemical properties of the amino acid residue at position 14 are highly conserved in bacterial, plant and vertebrate ferredoxins. This amino acid residue typically carries a positive charge required for protein stabilization through hydrogen bond formation with the conserved proline 108 residue (23). As zebrafish are the only teleost which retained both ferredoxin genes after the genome duplication of the common teleost ancestor (24) they provide a unique vertebrate model to further investigate the coevolution of ferredoxins and specific mitochondrial Cyp cofactors with regards to amino acid residue conservations, posttranscriptional regulation, stability, and electron transfer capabilities.

### Disruption of Fdx1b leads to glucocorticoid deficiency

Our expression analysis revealed *fdx1b* was restricted to the steroidogenic tissues, including high expression in the interrenal gland (imbedded within the kidney head). Additionally, *fdx1b* was only expressed during late larval stages once the interrenal gland had formed. This expression mirrors that of the zebrafish *cyp11a2*, the gene encoding P450 side-chain cleavage enzyme, which facilitates the rate-limiting step in steroidogenesis from 28 hpf (16). Therefore, we hypothesized Fdx1b is specifically regulating mitochondrial steroid hormone biosynthesis through electron transfer in the interrenal gland.

The use of TALENs has become a powerful and robust method in reverse genetics to study gene functions in zebrafish (25, 26). By employing TALENs to disrupt the open-reading frame of *fdx1b* we were able to generate zebrafish mutant alleles within the evolutionary conserved motif 1. Subsequently, 2 mutant lines were used to investigate the requirement of Fdx1b on steroidogenesis. The *fdx1b^UOB205^* allele has a 12-bp in-frame deletion, which removed a cysteine amino acid vital for Fe/S stabilization. It was anticipated that this disruption would generate a nonfunctional protein as mutation of any cysteine required for Fe/S interaction abolishes ferredoxin activity in vitro (6). The *fdx1b^UOB203^* allele resulted in a frame shift mutation that removed conserved motifs 2 and 3 of the protein. This mutation was also predicted to be deleterious and was therefore used for complimentary studies.

Homozygous *fdx1b* mutant larvae were identified by their disrupted VBA response, which has been reported in glucocorticoid-deficient (27) and glucocorticoid-resistant zebrafish larvae (28). Zebrafish are capable of background adaptation, which involves the distribution or aggregation of melanin within the melanophore to blend into the surrounding environment (29). This rapid and reversible physiological process is functional from 96 hpf and is regulated by glucocorticoid receptor signaling in teleost fish (20). Our results support this finding as *fdx1b* homozygous mutant larvae had impaired VBA, which was restored after incubation with the synthetic glucocorticoid receptor agonist dexamethasone. This suggested impaired glucocorticoid signaling in *fdx1b* mutants, which was confirmed by liquid chromatography tandem mass spectrometry cortisol measurements.

Human patients with adrenal insufficiency have an up-regulation of the hypothalamus-pituitary-adrenal axis with subsequent increased expression of *POMC*. Likewise, *fdx1b* mutants had an up-regulation of the HPI axis as indicated by increased *pomca* expression. This is consistent with other zebrafish models of glucocorticoid resistance leading to a disruption of the negative HPI feedback loop (27, 30). Expression of glucocorticoid response genes *pck1* and *fkbp5* were also decreased in *fdx1b^UOB205^* homozygous mutants under normal conditions supporting impaired glucocorticoid signaling. To further validate the model, the animals were subjected to osmotic stress, as this has been proven to increase cortisol production (17). However, there was no increase in cortisol or changes in the expression of glucocorticoid responsive genes in stressed *fdx1b* homozygous mutants. Overall, steroid hormone analysis supported by impaired VBA, altered HPI axis and decreased expression of cortisol responsive genes suggests that Fdx1b is the key mitochondrial redox protein involved in glucocorticoid biosynthesis in zebrafish.

### Fdx1b is required for glucocorticoid biosynthesis during early zebrafish development

The generation of *fdx1b* mutant zebrafish provides a useful tool to investigate the role of Fdx1b in steroid hormone biosynthesis and action. However, in order to investigate the requirement of Fdx1b in early development, a morpholino knockdown approach was used. Microinjections of morpholinos allow for large amounts of *fdx1b*-deficient embryos to be generated for biochemical analysis before the light adaptation phenotype becomes apparent. Cortisol measurements were assessed across development from *fdx1b* morphants and controls. Both control and *fdx1b*-deficient embryos depleted maternal cortisol stores after 24 hpf. After de novo cortisol biosynthesis begins at 48 hpf (13), we observed cortisol production in control larvae only. The return of cortisol biosynthesis at 96 and 120 hpf in *fdx1b*-deficient larvae is likely due to the transient nature of morpholinos. At 72 hpf gene disruption was confirmed in *fdx1b*-Spl^MO^-injected larvae and a decrease in cortisol in these larvae support the requirement of Fdx1b for glucocorticoid production. Furthermore, the cortisol deficiency detected from *fdx1b* morphants is consistent with our TALEN generated *fdx1b* mutants and with the knockdown of zebrafish P450scc enzyme, Cyp11a2 (16), and 11β-hydroxylase enzyme, Cyp11c1 (previously referred to as Cyp11b1 or Cyp11b) (31, 32)

### Fdx1b is the redox cofactor required for interrenal steroidogenesis

Adult zebrafish have 2 mitochondrial Cyp enzymes required for interrenal glucocorticoid production: Cyp11a2 (16) and Cyp11c1 (31). The mitochondrial P450scc enzyme Cyp11a2 is required for the first step of steroidogenesis, facilitating the biosynthesis of pregnenolone (16). Taking into consideration that only *fdx1b* transcripts were observed in the interrenal, we would expect reduced concentrations of the common steroid precursor pregnenolone in Fdx1b-deficient larvae. Despite consistently detecting lower concentrations of pregnenolone in *fdx1b* mutants and morphants when compared controls, these changes were not significant.

In vivo, pregnenolone has a low steady state as it is rapidly metabolized by 3β-hydroxysteroid dehydrogenase (Hsd3b) and steroid 17α-hydroxylase (Cyp17a1). Thus, we speculate that this might make changes in intermediate steroid metabolites poorly sensitive to ferredoxin modifications. Therefore, we used 11-deoxycortisol as an indirect measure of interrenal pregnenolone production. Given our limited sample volume and low concentration of steroid metabolites, we were unable to reliably detect other steroid hormone intermediates with this method. The detection of the 11-deoxycortisol and cortisol in control larvae confirmed pregnenolone was being metabolized by the zebrafish interrenal-specific enzymes Hsd3b1 (27) and 17α-hydroxylase 2 (Cyp17a2). In contrast, the decreased 11-deoxycortisol concentrations in *fdx1b*-deficient larvae suggest pregnenolone is not available for metabolism by steroidogenic enzymes for glucocorticoid biosynthesis. This supports impaired interrenal Cyp11a2 activity in Fdx1b-deficient larvae. Currently, it is unclear why *fdx1b*-deficient larvae have a detectable pregnenolone pool. Previous studies have shown pregnenolone is synthesized by Cyp11a1 in the yolk syncytial layer during early embryonic stages (33). However, we hypothesize the pregnenolone detected in *fdx1b* morphants and mutants could be a residual from embryonic steroid biosynthesis by Cyp11a1. Importantly, incubation with pregnenolone restored 11-deoxycortisol concentrations in *fdx1b*-deficient larvae. Thus confirming when pregnenolone is available it can be used for interrenal steroidogenesis and subsequent 11-deoxycortisol biosynthesis. Taken together with our expression analysis, we suggest Fdx1b is the main redox partner to the mitochondrial CYP enzyme Cyp11a2 within the zebrafish interrenal gland.

In humans, 11-deoxycortisol is converted to cortisol by the mitochondrial 11β-hydroxylase enzyme, CYP11B1, which also requires FDX1 electron transfer. Human *CYP11B* genes evolved from the ancestral *cyp11c1* of fish (34) and *cyp11c1* is the only identified Cyp11b-like enzyme in zebrafish (35). Like human CYP11B1, zebrafish Cyp11c1 is required for cortisol production during zebrafish development (31). To establish the requirement of Cyp11c1 on electron transport from Fdx1b, we incubated *fdx1b* morphants with pregnenolone, which has been proven to increase cortisol production in Cyp11a2-deficient larvae (16). At this time point, larvae can synthesize cortisol de novo independent of HPI regulation (36). Despite pregnenolone conversion to 11-deoxycortisol, Fdx1b morphants failed to produce cortisol, suggesting a crucial role of Fdx1b as an electron providing cofactor in regulating the final enzymatic step of glucocorticoid biosynthesis through Cyp11c1 activity.

### Zebrafish Fdx1 is required for early development

In concordance with human *FDX1* (37, 38), analysis of the zebrafish *fdx1* revealed that it was expressed throughout development and was widely expressed in adults. It is expected that Fdx1 is required for the activity of other nonsteroidogenic CYP enzymes such as those required for bile acid and vitamin D synthesis (39). Knockdown of *fdx1* showed a strong cellular defect with delayed epiboly. In comparison, knockdown of the maternally expressed P450scc enzyme Cyp11a1 (33) and Hsd3b2 (27) both reveal similar epiboly defects. Together, this suggests a potential role for Fdx1 as the redox partner of Cyp11a1 during early zebrafish development. Importantly, overexpression of *fdx1b* and *fdx1* could restore the loss of *fdx1* phenotype, signifying functional conservation between the 2 paralogues. Furthermore, it highlights the redox regulation is important for early morphological developments beyond its impact on steroidogenesis and warrants further investigation.

In summary, we have used a combination of genetic engineering and antisense knockdown approaches to study for the first time the impact of mitochondrial electron transfer regulation of glucocorticoid biosynthesis in vivo. Through molecular and biochemical approaches we characterized the role of Fdx1 and Fdx1b during zebrafish development. By using TALENs, we have successfully generated *fdx1b* mutant lines, permitting disruption of the zebrafish steroid biosynthesis pathway, which was reflected as glucocorticoid deficiency during early development. Due to the absence of the gonad at these developmental stages, the impact on gonadal steroidogenesis warrants future studies. Furthermore, our identification of Fdx1b as the key regulator of mitochondrial steroidogenesis in the zebrafish interrenal gland has allowed us to specifically investigate steroidogenic mitochondrial redox regulation without disruption of early zebrafish development or other mitochondrial redox regulated processes. Our *fdx1b* mutants represent the first in vivo knockout model system for a ferredoxin gene. This zebrafish model will provide a valuable resource for studying the impact of glucocorticoid production by mitochondrial redox reactions and will give insights into the role of mitochondrial electron transfer in other processes, including development, stress research, and behavior.
